# Head-to-head comparison of neurodegeneration biomarkers across two analytical platforms in Alzheimer’s disease

**DOI:** 10.1007/s40520-026-03420-5

**Published:** 2026-06-25

**Authors:** Carolin Kurz, Daniela Hattenkofer, Maximilian Pihale-Haug, Anna Hufnagel, Selim Üstün Gürsel, Matthias Brendel, Boris-Stephan Rauchmann, Johannes Levin, Günter Höglinger, Robert Perneczky

**Affiliations:** 1 Department of Psychiatry and Psychotherapy, LMU Hospital LMU Munich, 80336 Munich, Germany; 2 German Center for Neurodegenerative Diseases (DZNE), 81377 Munich, Germany; 3 Department of Nuclear Medicine, LMU Hospital LMU Munich, 81377 Munich, Germany; 4 Munich Cluster for Systems Neurology (SyNergy), 80336 Munich, Germany; 5 Department of Radiology, LMU Hospital LMU Munich, 81377 Munich, Germany; 6 Department of Neurology, LMU Hospital LMU Munich, 81377 Munich, Germany; 7 Department of Neuroradiology, LMU Hospital, LMU Munich, 81377 Munich, Germany; 8 Ageing Epidemiology (AGE) Research Unit, School of Public Health, Imperial College London, London, UK; 9 International Registry for Alzheimer’s, Disease and Other Dementias (InRAD) Foundation, Deventer, Netherlands

**Keywords:** Alzheimer disease/blood, Biomarkers/blood, Glial fibrillary acidic protein/blood, pTau217/blood, Blood-based biomarkers, Neurodegeneration, Inter-test agreement

## Abstract

**Background:**

Blood-based biomarkers are increasingly used to support the identification of Alzheimer’s disease (AD) in clinical and research settings. However, variability between analytical platforms limits their interchangeability and may affect their clinical interpretation. Direct head-to-head comparisons across assays and their alignment with clinical phenotypes remain limited.

**Objective:**

To compare the inter-test agreement of blood-based biomarkers measured using Roche and Fujirebio assays and to determine which biomarker shows the most robust analytical concordance and clinical relevance in AD.

**Methods:**

Participants with AD (n = 86) and cognitively healthy controls (HC; n = 56) underwent blood biomarker assessment using Roche and Fujirebio platforms. Plasma phosphorylated tau at threonine 217 (pTau217), plasma phosphorylated tau at threonine 181 (pTau181), Amyloid-β42 (Aβ42), amyloid-β40 (Aβ40), and the amyloid-β42/amyloid-β40 ratio (Aβ42/Aβ40) and glial fibrillary acidic protein (GFAP) were analyzed. Inter-test agreement was assessed using intraclass correlation coefficients, concordance analyses, and regression-based methods. Clinical relevance was evaluated by association with measures of cognitive and functional impairment evaluated using the Mini-Mental State Examination (MMSE) and the Clinical Dementia Rating Scale – Sum of Boxes (CDR-SB). Analyses were adjusted for age and sex and complemented by sensitivity and stability analyses.

**Results:**

Inter-test agreement varied across biomarkers, with pTau217 demonstrating the highest and most stable concordance between Roche and Fujirebio assays. Among the evaluated markers, pTau217 also showed the strongest and most consistent associations with global cognition measures across diagnostic groups and best discriminated between individuals with AD and HC. GFAP demonstrated robust inter-test agreement but weaker alignment with clinical measures, while amyloid-based markers showed lower concordance and greater variability across platforms.

**Conclusions:**

Head-to-head comparison of Roche and Fujirebio assays revealed biomarker-specific differences in inter-test agreement, limiting the direct interchangeability of measurements across platforms. Plasma pTau217 showed the most consistent analytical concordance and clinical associations in this setting, supporting its potential utility in cross-platform applications. However, these findings also highlight the need for assay-specific calibration and cautious interpretation of biomarker thresholds in clinical and research use and the need for further validation across diverse populations.

**Trial registration:**

NCT05059158, NCT05317871

**Supplementary Information:**

The online version contains supplementary material available at 10.1007/s40520-026-03420-5.

## Background

Blood-based biomarkers are increasingly integrated into the diagnostic work-up of neurodegenerative diseases, particularly Alzheimer’s disease (AD). [1] Their accessibility, scalability, and potential for early disease detection make them attractive tools for both clinical practice and large-scale research studies. [2, 3] AD represents a particularly suitable „model disease“ in this context, as several disease-associated pathological processes can be detected through specific protein alterations in peripheral blood.

Among the most established AD-related blood biomarkers are amyloid-β42 (Aβ42) and amyloid-β40 (Aβ40), typically expressed as the amyloid-β42/amyloid-β40 ratio (Aβ42/Aβ40), as well as phosphorylated tau species such as phosphorylated tau181 (p-tau181) and phosphorylated tau1217 (p-tau217). [4] These biomarkers reflect core pathological processes of AD, including amyloid plaque deposition and tau pathology, and are therefore considered relatively disease-specific indicators of AD-related neurodegeneration. [5, 6].

In contrast, other blood biomarkers such as neurofilament light chain (NfL) and glial fibrillary acidic protein (GFAP) are not considered disease-specific. [7, 8] Rather, they are thought to reflect more general processes of neuronal injury and astroglial activation. As such, these markers may function as dynamic indicators of disease stage, progression, or prognosis across different neurodegenerative conditions, rather than as specific markers of AD pathology. [9] This distinction highlights the complementary roles of biomarkers that primarily capture disease-defining pathology (e.g., Aβ and p-tau) and those that may provide information about the intensity or temporal dynamics of neurodegeneration (e.g., NfL and GFAP).

Despite their increasing use, blood-based biomarkers are measured using different analytical platforms and assays, which may vary in calibration, analytical sensitivity, and dynamic range. Such inter-assay variability challenges the direct comparability and interchangeability of biomarker results across centers and studies. This limitation is particularly relevant in multicenter settings and longitudinal cohorts, where changes in assay platforms over time may confound clinical interpretation.

While several studies have demonstrated the diagnostic performance of individual blood-based biomarkers, direct head-to-head comparisons between commonly used assay platforms remain scarce. [10] Moreover, most existing comparisons focus on analytical correlations alone, without systematically assessing whether inter-test agreement translates into comparable clinical relevance. It therefore remains unclear which biomarkers combine robust analytical concordance across platforms with meaningful alignment to clinical phenotypes across the AD spectrum.

Assessing biomarker agreement not only at the analytical level but also against clinical outcomes may better reflect their utility in real-world diagnostic settings.

The aims of this study were (i) to perform a head-to-head comparison of Roche and Fujirebio assays for key blood-based biomarkers and quantify their inter-test agreement, and (ii) to determine whether analytical concordance translates into consistent clinical associations across platforms. By explicitly combining analytical and clinical validation, this study aims to provide a comprehensive assessment of cross-platform applicability in real-world settings.

## Methods

We conducted a secondary pooled analysis of two prospective observational cohort studies performed at LMU Hospital Munich: The AmyClear study (NCT05059158, registered 2021–09-16) and the PeptiClear study (NCT05317871, registered 2022–03-31), which followed harmonized protocols for clinical assessment, neuropsychological testing, neuroimaging, and biomarker sampling. Both studies were approved by the institutional ethics committee of LMU Munich (EC Nos. 18–0606 and 19–148), and all participants provided written informed consent in accordance with the Declaration of Helsinki. Plasma biomarker measurements using Roche assays were available in the overall cohort, whereas Fujirebio measurements were available only in a subset of participants from the AmyClear study. Consequently, cross-platform analyses (Roche vs Fujirebio) were restricted to this subset with paired plasma measurements. Pooling of the cohorts was justified as both studies were conducted within the same academic memory clinic, followed harmonized recruitment procedures, and used identical clinical, neuropsychological, imaging, biofluid, and pre-analytical protocols. The combined design enabled a direct comparison across AD and cognitively healthy controls within a unified analytical framework.

### Participants and diagnostic classification

Participants were classified into diagnostic groups according to guideline-based clinical criteria. For the present analyses, the primary contrast of interest was Alzheimer’s disease (AD) versus cognitively healthy controls (HC). AD cases included individuals with mild cognitive impairment due to Alzheimer’s disease as well as probable Alzheimer’s dementia, while cognitively healthy controls demonstrated normal cognitive performance and no evidence of neurodegenerative disease. Diagnoses were established by board-certified neurologists or psychiatrists specialized in dementia according to ICD-10 criteria and the German S3 guideline and supported by clinical, neuropsychological, and biomarker information where available.

Participants were assigned to two diagnostic groups:

1. Alzheimer’s disease (AD spectrum; including mild cognitive impairment due to AD and probable AD dementia) and
2. Cognitively healthy controls (HC).

AD-spectrum cases fulfilled National Institute on Aging–Alzheimer’s Association (NIA–AA) criteria supported by cerebrospinal fluid (CSF) and/or positron emission tomography (PET) biomarkers indicative of amyloid and tau pathology. Amyloid positivity was defined as either a positive amyloid PET scan (≥ 20 Centiloids) or a positive CSF Aβ42/Aβ40 ratio (≤ 0.055), determined using enzyme-linked immunosorbent assays (INNOTEST, Fujirebio). [11] In case of discordant results, participants were classified as amyloid positive if either biomarker indicated amyloid pathology, consistent with the NIA-AA research framework. [12].

Cognitively healthy controls demonstrated normal cognitive performance and biomarker-negative CSF profiles. All participants underwent harmonized clinical assessments, comprehensive neuropsychological testing, structural and molecular neuroimaging, and blood biomarker sampling. Detailed inclusion and exclusion criteria are provided in the Supplementary Material (Table S1).

### Clinical, neuropsychological, functional and imaging assessments

Clinical disease severity was assessed by trained and experienced board-certified physicians using standardized cognitive, functional, and imaging measures. Global cognition was evaluated using the Mini-Mental State Examination (MMSE) and the Clinical Dementia Rating Scale – Sum of Boxes (CDR-SB). [13, 14].

### Biomarker assessment

Venous blood samples were collected and processed according to standardized pre-analytical procedures. Plasma was separated, aliquoted, and stored at − 80°C until analysis. Plasma biomarkers were measured using two automated immunoassay platforms. Fujirebio Lumipulse® G assays were used for pTau217, pTau181, GFAP, and Aβ42/40, while Roche Elecsys® assays (Cobas e801 platform) were used for NfL and for cross-platform comparison of selected biomarkers where available. All measurements were performed on aliquots derived from the same blood samples. Samples were stored at − 80°C and were not previously thawed prior to analysis; all assays were performed after a single thaw cycle under standardized conditions. All samples were analyzed in a single batch per platform to minimize technical variability, and laboratory personnel were blinded to clinical and imaging data. Assay calibration and quality control procedures followed manufacturer recommendations.

Previously published reference thresholds for plasma biomarkers measured with the Fujirebio Lumipulse platform were used for contextual interpretation of biomarker concentrations. Reported thresholds for pTau217 include a two-cut-off approach (< 0.37 pg/mL indicating low probability and > 0.59 pg/mL indicating high probability of amyloid pathology) as well as a single cut-off of approximately 0.49 pg/mL derived from amyloid PET–based classification. [1, 2] For pTau181, a threshold of approximately > 2.0 pg/mL has been suggested for distinguishing Alzheimer’s disease from cognitively normal individuals, while an Aβ42/40 ratio below approximately 0.08 has been associated with Alzheimer-type pathology. [15.16] Plasma NfL concentrations were interpreted using age-dependent reference ranges, reflecting their known increase with age. A summary of previously reported biomarker reference thresholds is provided in Table S2. [15].

### Statistical analysis

The statistical analysis comprised two predefined modules. First, inter-assay agreement of plasma biomarkers measured on Roche and Fujirebio platforms was evaluated to identify the biomarker with the most robust analytical concordance and to derive diagnostic cut-offs for biomarker-defined amyloid status. Agreement was assessed using intraclass correlation coefficients for absolute agreement, Spearman correlations, Passing–Bablok regression, and Bland–Altman analyses. Passing–Bablok regression was used as a robust non-parametric method for method comparison. Stability of agreement estimates was evaluated using bootstrap resampling. Amyloid positivity was defined as either a positive amyloid PET scan (≥ 20 Centiloids) or a pathological CSF Aβ42/Aβ40 ratio (≤ 0.055). Discriminative performance was assessed using receiver operating characteristic (ROC) analyses, with area under the curve (AUC) and 95% confidence intervals. Optimal Roche cut-offs were determined using a sensitivity-weighted Euclidean distance approach and are reported as exploratory. Cross-platform comparability was assessed in participants with paired plasma measurements using correlation and regression-based bridging analyses. Linear regression analyses indicated a stable and approximately linear relationship between platforms and were used as a pragmatic approach to enable direct translation of biomarker cut-offs between assays. In contrast to Passing–Bablok regression, which was applied for method comparison, linear regression was specifically used here for calibration purposes. Published Fujirebio cut-offs were translated to the Roche scale using linear regression models (log-transformed where appropriate). Second, associations between the most analytically stable biomarker and cognitive performance were evaluated using correlation analyses and linear regression models adjusted for age, sex, and education. All analyses were conducted in R (version 2025.05.1). Skewed variables were log-transformed where appropriate. Multivariate outliers were identified using Mahalanobis distance (p < 0.001) and excluded. Multiple testing was controlled using the Benjamini–Hochberg false discovery rate across all primary analyses, with two-sided p < 0.05 considered statistically significant.

## Results

**Fig. 1:**
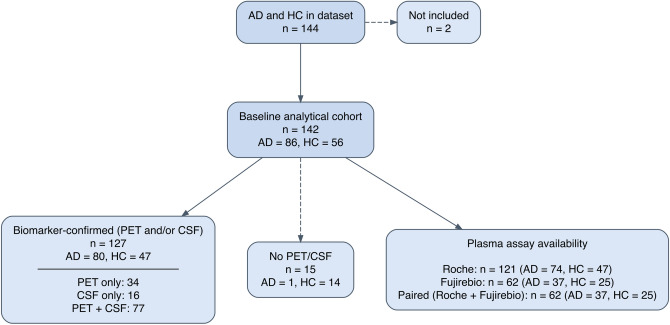
Participant flow and selection of the final biomarker-confirmed cohort. Flow diagram illustrating participant selection and derivation of the final biomarker-confirmed study cohort. Abbreviations: AD, Alzheimer’s disease; HC, healthy controls; CSF, cerebrospinal fluid; PET, Positron emission tomography

A total of 144 participants were included in the dataset, of whom 127 had biomarker confirmation at baseline and constituted the final study cohort (AD: n = 80; HC: n = 47), as illustrated in Fig. 1. All subsequent analyses refer to this cohort unless otherwise specified. Slight variations in sample size across analyses reflect differences in data availability for specific biomarkers or modalities (e.g., CSF, paired plasma measurements) and are reported in the respective tables and figure legends. Biomarker confirmation was defined as the availability of either cerebrospinal fluid (CSF) biomarkers or amyloid PET imaging. Baseline assessments were available for all participants included in the final cohort. All p-values reported in the Results section correspond to FDR-corrected values where applicable.

### Reference to previously published cohort and primary findings

Participants were recruited consecutively from an academic memory clinic (LMU Hospital, Munich) between 2021 and 2025**.** After screening for predefined inclusion and exclusion criteria by study physicians (Supplementary Table S1), eligible patients were actively approached and invited to participate.

Baseline characteristics of the biomarker-confirmed cohort are summarized in Table 1, broadly reflecting a typical research cohort with predominantly early-stage AD and mild clinical impairment. AD participants were older than HC (72 ± 8 vs 68 ± 8 years; p = 0.005). There were no significant group differences in years of education, body mass index, or depressive symptoms. As expected, AD participants showed greater clinical impairment (CDR-SoB: 2.7 ± 2.0 vs 0.5 ± 1.0; p < 0.001) and lower global cognition (MMSE: 24.9 ± 3.9 vs 28.6 ± 3.5; p < 0.001). APOE ε4 carriership was more frequent in AD than HC. Detailed demographic and clinical characteristics are provided in Table 1.

**Table 1:** Baseline demographic and clinical characteristics (AD vs HC)

Characteristic	HC	AD	P	P (FDR)
n (baseline)	61	81		
Age at baseline, years	67.7 (7.9)	71.8 (8.5)	0.005	0.009
Education, years	11.2 (16.3)	12.8 (3.3)	0.101	0.162
Body mass index, kg/m^2^	26.0 (4.2)	25.3 (3.8)	0.212	0.282
CDR-SoB	0.4 (1.0)	2.7 (2.2)	< 0.001	< 0.001
MMSE	28.5 (3.7)	24.8 (4.5)	< 0.001	< 0.001
GDS	2.1 (2.2)	2.1 (2.2)	0.596	0.596
Sex: Female	32 (46.4%)	37 (44.6%)	0.384	0.439
Sex: Male	37 (53.6%)	46 (55.4%)		
APOE ε4 status: Missing	4 (7.1%)	10 (11.6%)	< 0.001	< 0.001
APOE ε4 status: Non-carrier	39 (69.6%)	25 (29.1%)		
APOE ε4 status: ε4 heterozygous	11 (19.6%)	41 (47.7%)		
APOE ε4 status: ε4 homozygous	2 (3.6%)	10 (11.6%)		
Biomarker availability				
Plasma assays				
Roche available, n (%)	47 (83.9%)	74 (86.0%)		
Fujirebio available, n (%)	25 (44.6%)	37 (43.0%)		
Amyloid PET available, n (%)	40 (71.4%)	71 (82.6%)	0.146	0.244
CSF available, n (%)	35 (62.5%)	58 (67.4%)	0.590	0.590
Both PET and CSF available, n (%)	28 (50.0%)	49 (57.0%)	0.491	0.590
Amyloid PET Centiloid	-12.1 (9.3)	70.3 (30.0)	< 0.001	< 0.001
Amyloid PET status (cutoff 20 CL)				
Aβ negative, n (%)	40 (100.0%)	5 (7.0%)	< 0.001	< 0.001
Aβ positive, n (%)	0 (0.0%)	66 (93.0%)		
CSF Aβ status (ratio ≤ 5.5)				
Aβ negative (%)	35 (100.0%)	0 (0.0%)	< 0.001	< 0.001
Aβ positive (%)	0 (0.0%)	58 (100.0%)		
Overall Biomarker-defined Aβ status (CSF or PET)				
Aβ negative (%)	35 (100.0%)	0 (0.0%)	< 0.001	< 0.001
Aβ positive (%)	0 (0.0%)	78 (100.0%)		

Continuous variables are reported as mean (SD). Categorical variables are reported as n (%), including missing values. P-values are based on Wilcoxon rank-sum tests (continuous) and Fisher’s exact tests (categorical) with Benjamini–Hochberg FDR correction. Abbreviations: AD, Alzheimer’s disease; HC, healthy controls; BMI, body mass index; CDR-SoB, Clinical Dementia Rating Sum of Boxes; MMSE, Mini-Mental State Examination; GDS, Geriatric Depression Scale; APOE, apolipoprotein E; CSF, cerebrospinal fluid; PET, positron emission tomography; Aβ, amyloid beta; CL, Centiloid. Sample sizes may differ slightly from the final analytical cohort due to missing data in specific variables

At baseline, 56 cognitively healthy participants with subjective cognitive impairment (HC/SCI) and 86 patients with Alzheimer’s disease (AD) were included in the plasma biomarker analyses. Plasma biomarkers measured with the Roche platform showed no significant group difference for Aβ1-40 or Aβ1-42 concentrations (Aβ1-40: HC 0.215 ± 0.068 ng/mL vs AD 0.225 ± 0.066 ng/mL, p = 0.496; Aβ1-42: HC 26.651 ± 9.907 pg/mL vs AD 24.330 ± 9.726 pg/mL, p = 0.159). However, the plasma Aβ42/40 ratio was significantly lower in AD compared with HC (0.124 ± 0.025 vs 0.107 ± 0.020, p < 0.001). In addition, plasma concentrations of GFAP, neurofilament light (NfL), p-tau217, and p-tau181 were significantly higher in the AD group than in HC (all p < 0.001). Complete biomarker concentrations are summarized in Table 2.

**Table 2:** Baseline plasma and CSF biomarker concentrations (AD – HC)

Characteristic	HC, mean (SD)	AD, mean (SD)	P	P (FDR)
*Roche plasma biomarkers*				
N (Baseline)	47	74		
Aß1–40 (NG/ML)	0.215 (0.068)	0.225 (0.066)	0.496	0.527
Aß1–42 (PG/ML)	26.651 (9.907)	24.330 (9.726)	0.159	0.193
Aß42/40 RATIO	0.124 (0.025)	0.107 (0.020)	< 0.001	< 0.001
GFAP (NG/ML)	0.093 (0.056)	0.146 (0.066)	< 0.001	< 0.001
NFL (PG/ML)	2.398 (2.385)	2.897 (1.465)	< 0.001	< 0.001
P-TAU217 (PG/ML)	0.123 (0.067)	0.421 (0.285)	< 0.001	< 0.001
P-TAU181 (PG/ML)	0.776 (0.260)	1.462 (0.734)	< 0.001	< 0.001
*Fujirebio plasma biomarkers*				
N	25	37		
Aß1–40 (PG/ML)	163.724 (73.015)	153.474 (60.375)	< 0.001	< 0.001
Aß1–42 (PG/ML)	16.562 (6.897)	12.334 (4.664)	0.013	0.017
Aß42/40 RATIO	0.103 (0.011)	0.081 (0.013)	< 0.001	< 0.001
GFAP (PG/ML)	50.748 (27.910)	90.400 (83.039)	< 0.001	< 0.001
P-TAU217 (PG/ML)	0.102 (0.041)	0.552 (0.406)	< 0.001	< 0.001
*CSF biomarkers (innotest)*				
N	35	58		
Aß1–40 (PG/ML)	14,031.229 (5759.418)	13,647.328 (5809.578)	0.493	0.527
Aß1–42 (PG/ML)	1142.074 (565.501)	497.109 (194.540)	< 0.001	< 0.001
Aß42/40 RATIO	0.086 (0.037)	0.042 (0.041)	< 0.001	< 0.001
TOTAL TAU (PG/ML)	240.632 (94.531)	456.301 (205.170)	< 0.001	< 0.001
P-TAU181 (PG/ML)	51.603 (14.637)	92.084 (33.865)	< 0.001	< 0.001

Continuous variables are presented as mean (SD). Group differences were assessed using Wilcoxon rank-sum tests with Benjamini–Hochberg false discovery rate (FDR) correction. Plasma Aβ42/40 ratios were calculated as Aβ1-42 divided by Aβ1-40 using concentrations measured by the respective assay platform (Roche or Fujirebio). CSF Aβ42/40 ratio was calculated from CSF Aβ1-42 and Aβ1-40 concentrations. HC denotes cognitively healthy and amyloid negative individuals with subjective cognitive impairment. Units differ across assays and are reported as provided by the manufacturer. The reported sample sizes reflect availability of plasma biomarker data and may differ from the overall analytical cohort

Similarly, plasma biomarkers measured with the Fujirebio platform demonstrated significantly lower Aβ1-42 concentrations and a significantly lower Aβ42/40 ratio in AD compared with HC, whereas Aβ1-40 levels showed no consistent difference between groups. Plasma GFAP and p-tau217 concentrations measured by Fujirebio were markedly increased in AD compared with HC. Detailed results are provided in Table 2.

CSF biomarker data were available for a subset of participants (HC: n = 35; AD: n = 58). CSF Aβ1-40 concentrations did not differ between groups (14,031 ± 5759 vs 13,647 ± 5809 pg/mL, p = 0.493), whereas CSF Aβ1-42 concentrations and the CSF Aβ42/40 ratio were significantly lower in AD compared with HC. In contrast, CSF total tau and CSF p-tau181 levels were significantly higher in AD patients (all p < 0.001). Detailed CSF biomarker results are summarized in Table 2.

**Fig. 2:**
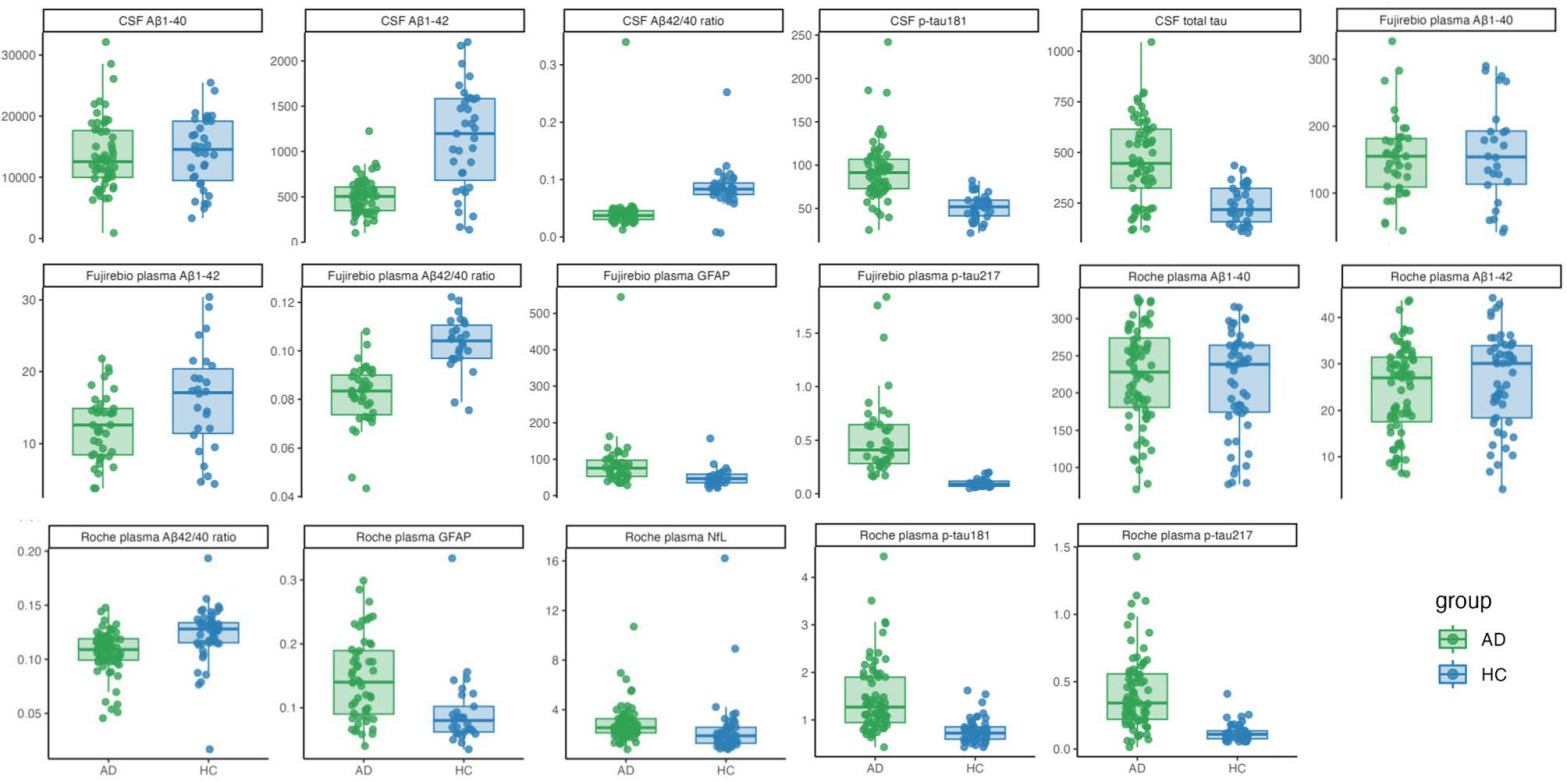
Distribution of plasma and CSF biomarker concentrations (AD vs. HC). Inter-assay agreement between plasma biomarker measurements obtained using the Roche and Fujirebio platforms. Scatterplots display paired measurements for (**A**) p-tau217, (**B**) the Aβ42/40 ratio and (**C**) GFAP. Each point represents an individual participant. Solid lines indicate linear regression fits, illustrating the relationship and degree of concordance between the two assay platforms

The distribution of biomarker concentrations in HC and AD is illustrated in Fig. 2. Across both plasma platforms, the strongest separation between groups was observed for phosphorylated tau biomarkers, particularly plasma p-tau217, followed by the Aβ42/40 ratio. Effect size estimates (Hedges’ g) for all biomarkers are summarized in Supplementary Table S4, highlighting the largest effects for p-tau217 and the Aβ42/40 ratio across assays.

### Inter-assay agreement across biomarkers

Inter-assay agreement analyses between the Roche and Fujirebio platforms are summarized in Table 3. Overall, the strongest cross-platform concordance was observed for plasma p-tau217 (ρ = 0.91; ICC = 0.91) and Aβ1-40 (ρ = 0.90; ICC = 0.88), whereas agreement for Aβ1-42 was moderate and lower for the Aβ42/40 ratio (ρ = 0.65; ICC = 0.38). Although plasma GFAP showed a very high correlation (ρ = 0.97), absolute agreement between platforms was low (ICC = 0.01), indicating systematic differences in measurement scale between assays. Comparisons between plasma and CSF biomarkers revealed generally modest concordance, with the strongest associations observed for the plasma Aβ42/40 ratio, while correlations for individual Aβ peptides were weak or absent. Overall, these findings indicate biomarker-specific differences in cross-platform comparability and limited interchangeability between peripheral and central amyloid measures. Additional agreement and plasma–CSF analyses are provided in Table 3 and the Supplementary Material.

**Table 3:** Inter-assay agreement and plasma–CSF concordance of biomarker measurements

		Inter-assay agreement (Roche vs Fujirebio)						
Comparison	N (paired)		HC	AD	Spearman Ρ	ICC	Bias (LOA)	Scale
Plasma Aß40	57		24	33	0.899	0.878	-0.063 (-0.194 to 0.069)	Log10
Plasma Aß42	59		24	35	0.808	0.646	-0.100 (-0.403 to 0.203)	Log10
Plasma Aß42/40 ratio	57		24	33	0.645	0.379	-0.011 (-0.055 to 0.033)	raw
Plasma GFAP	19		5	14	0.974	0.014	2.720 (2.595 to 2.844)	Log10
Plasma p-tau217	62		25	37	0.914	0.912	-0.022 (-0.335 to 0.290)	Log10
*Plasma–CSF concordance (Aβ-related biomarkers only)*								
Aß42/40 ratio	89		35	58	0.407	0.098	0.054 (-0.034 to 0.142)	raw
Aß40	89		35	58	-0.039	-0.000	-1.765 (-2.288 to -1.241)	Log10
Aß42	91		36	55	0.002	0.002	-1.418 (-2.087 to -0.749)	Log10
*Fujirebio plasma assays vs CSF reference*								
Aß42	46		15	31	0.268	0.012	-1.721 (-2.273 to -1.169)	Log10
Aß40	46		15	31	0.164	0.002	-1.967 (-2.445 to -1.488)	Log10
Aß42/40 ratio	46		15	31	0.519	0.271	0.031 (-0.035 to 0.096)	raw

Spearman correlation coefficients, intraclass correlation coefficients (ICC; absolute agreement), and Bland–Altman statistics (bias and limits of agreement, LoA) are reported for inter-assay comparisons and plasma–CSF concordance analyses. Inter-assay agreement was assessed between Roche and Fujirebio plasma measurements. Plasma–CSF concordance analyses were restricted to Aβ-related biomarkers, as corresponding CSF reference measures were not available for other plasma biomarkers (e.g., p-tau217, GFAP, NfL). ICC and Bland–Altman analyses were performed on log10-transformed values where indicated, whereas Spearman correlations were calculated on raw paired measurements

### Regression-based agreement and systematic bias

**Fig. 3:**
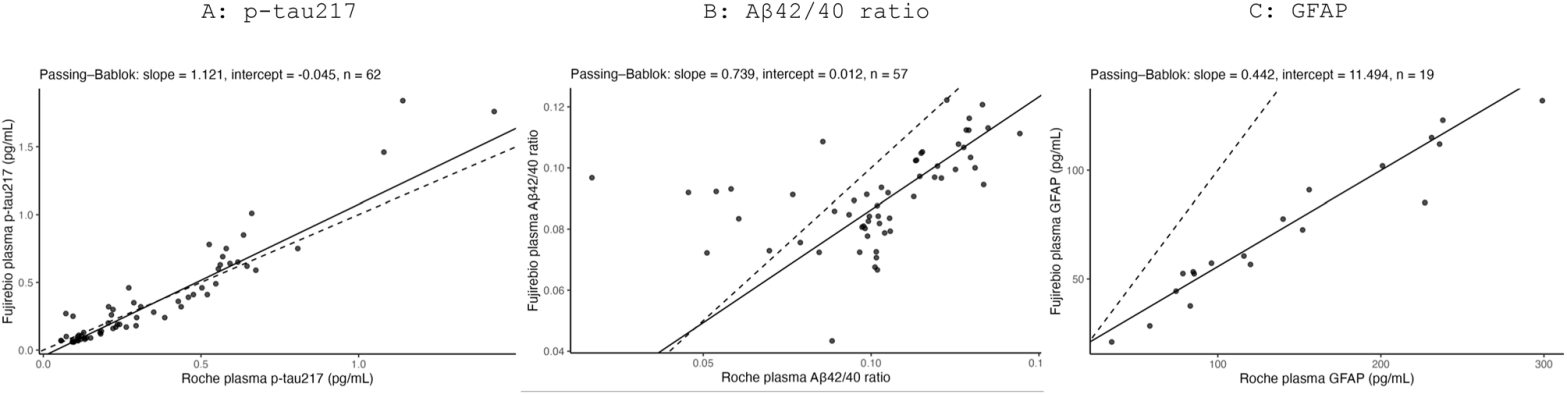
Inter-assay agreement between Roche and Fujirebio plasma assays. Bland–Altman plots illustrating limits of agreement (LoA) between Roche and Fujirebio plasma assays for (**A**) p-tau217, (**B**) the Aβ42/40 ratio and and (**C**) GFAP. Differences between paired measurements are plotted against their mean values. The central solid line represents the mean difference (bias), while dashed lines indicate the limits of agreement (± 1.96 standard deviations). These plots assess systematic bias and variability between assay platforms

Regression-based agreement analyses using Passing–Bablok regression demonstrated slopes close to unity for several biomarkers with strong cross-platform concordance. For plasma p-tau217, the regression slope was 1.12 with an intercept of − 0.05 (n = 62), indicating minimal proportional or constant bias between the Roche and Fujirebio platforms (Fig. 3A). Similarly, plasma Aβ1-40 showed a slope close to unity (1.10), consistent with good cross-platform agreement. In contrast, biomarkers with lower concordance exhibited greater deviations from the line of identity. Plasma Aβ1-42 demonstrated a slope below unity (0.84) with a negative intercept (− 1.35; n = 59), suggesting systematic differences between platforms across the measurement range (Fig. 3B). The plasma Aβ42/40 ratio also showed a slope below unity (0.74), indicating proportional differences between assay platforms. For GFAP, Passing–Bablok regression demonstrated a substantially lower slope (0.44) with a positive intercept (11.49; n = 19), consistent with systematic scale differences between Roche and Fujirebio measurements despite the high correlation observed between assays (Fig. 3C). These regression analyses confirm the variability in cross-platform agreement observed in the correlation and ICC analyses (Table 3).

### Plasma–CSF concordance

Plasma–CSF comparisons were restricted to Aβ-related biomarkers due to the availability of corresponding CSF reference measures. Comparisons between plasma and CSF biomarkers revealed generally modest concordance (Table 3). The strongest plasma–CSF association was observed for the Aβ42/40 ratio measured with the Fujirebio assay (ρ = 0.52; ICC = 0.27), followed by the Roche assay (ρ = 0.41; ICC = 0.10). Bland–Altman analysis further demonstrated a mean bias of 0.05 with limits of agreement ranging from − 0.03 to 0.14 for the Roche assay (Fig. 3C). In contrast, correlations between plasma and CSF concentrations of individual amyloid peptides were weak or absent. Plasma Aβ1-42 showed minimal correlation with CSF concentrations, while plasma Aβ1-40 showed no meaningful association with CSF levels (Table 3). These findings indicate limited concordance between peripheral and central measures of individual amyloid peptides, whereas the plasma Aβ42/40 ratio demonstrated comparatively stronger correspondence with CSF amyloid status.

### Bland–Altman analysis

**Fig. 4:**
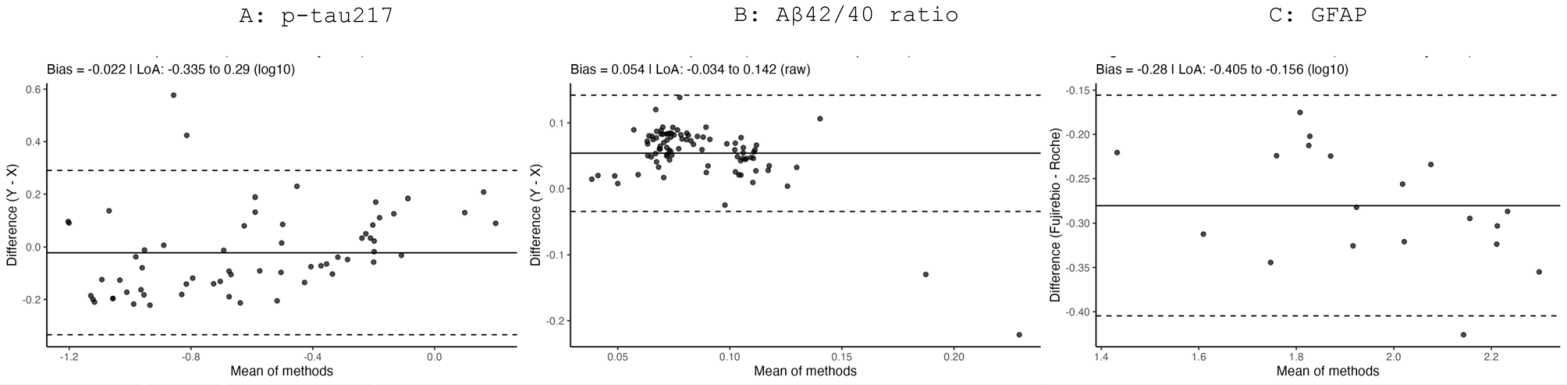
Limits of Agreement (LoA) measured by Bland–Altman analyses. Boxplots illustrate the distribution of biomarker concentrations measured in plasma (Roche and Fujirebio platforms) and cerebrospinal fluid (CSF) in cognitively healthy participants (HC) and patients with Alzheimer’s disease (AD). Boxes represent the interquartile range (IQR) with the median indicated by the central line; whiskers extend to 1.5 × IQR, and individual data points are shown. AD patients demonstrate lower Aβ42 concentrations and Aβ42/40 ratios, as well as higher concentrations of tau-related biomarkers (p-tau217 and p-tau181). Biomarker values are shown on their original measurement scales

Bland–Altman analyses were performed to assess absolute agreement and systematic measurement differences between assay platforms and biological compartments. For p-tau217 measured in plasma using Roche and Fujirebio assays, the mean bias was low (− 0.02 on the log10 scale), indicating minimal systematic offset between platforms. However, the relatively wide limits of agreement (− 0.34 to 0.29) suggest variability at the individual level. In contrast, plasma GFAP measurements demonstrated a substantially larger systematic offset between assay platforms, with a mean bias of approximately − 0.28 and limits of agreement ranging from − 0.41 to − 0.16 on the log10 scale (Fig. 4C), consistent with the observed scale-dependent differences between Roche and Fujirebio measurements. Comparison of the Aβ42/40 ratio between CSF and Roche plasma measurements demonstrated a mean bias of 0.05 and limits of agreement ranging from − 0.03 to 0.14, indicating greater variability between compartments and more limited interchangeability with CSF-derived ratios. Visual inspection of the Bland–Altman plots did not reveal strong evidence of heteroscedasticity or pronounced proportional bias after log-transformation where appropriate. The corresponding Bland–Altman plots for the primary biomarkers are presented in Fig. 4.

### Stability of inter-test agreement

Bootstrap-based stability analyses demonstrated that biomarkers with strong cross-platform concordance also exhibited robust agreement metrics across resampling iterations. Plasma p-tau217 and Aβ1-40 showed the highest stability, with agreement scores of 1.91 and 1.67, respectively (Supplementary Table S3), consistent with the strong concordance observed in the Passing–Bablok and Bland–Altman analyses (Figs. 3A-B and 4A-B). In contrast, biomarkers with lower baseline concordance showed greater variability across resampling iterations. For example, the plasma Aβ42/40 ratio demonstrated a negative agreement score (− 1.60), while GFAP showed the lowest score (− 2.28), indicating more limited robustness of cross-platform comparability and consistent with the greater dispersion and systematic assay-dependent differences observed for these biomarkers (Figs. 3B-C and 4B–C). However, the number of paired measurements available for GFAP was substantially smaller than for the other biomarkers (n = 19; Table 3), which may limit the interpretability of agreement estimates for this marker.

Sensitivity analyses further supported the robustness of the observed agreement patterns. Exclusion of influential observations did not materially alter correlation coefficients or regression parameters for the primary biomarkers. Similar stability was observed for Aβ1-42 and the Aβ42/40 ratio. In addition, stratified analyses by diagnostic group demonstrated comparable inter-assay correlations in both AD and HC participants, indicating that the observed agreement between assay platforms was not driven by disease-related differences in biomarker concentrations. Among all evaluated biomarkers, plasma p-tau217 achieved the highest agreement score, indicating the most consistent cross-platform concordance within the present dataset.

### Associations between plasma biomarkers and clinical severity

Associations between plasma biomarkers and baseline clinical severity were assessed using linear regression models adjusted for age, sex, and years of education (Figure S1; Table S5). Higher plasma p-tau217 concentrations measured with both the Roche and Fujirebio platforms were significantly associated with greater clinical severity and lower cognitive performance. Similar associations were observed for plasma GFAP concentrations, although associations for Fujirebio GFAP did not consistently reach statistical significance. Lower plasma Aβ42/40 ratios were also associated with greater clinical severity, consistent with more advanced disease. Overall, plasma p-tau217 showed the most consistent associations with both clinical severity measures across analytical platforms, followed by GFAP and the Aβ42/40 ratio. Detailed regression coefficients and confidence intervals are provided in Table S5.

### Cross-platform comparison and cut-off bridging between Roche and Fujirebio plasma biomarkers

**Fig. 5:**
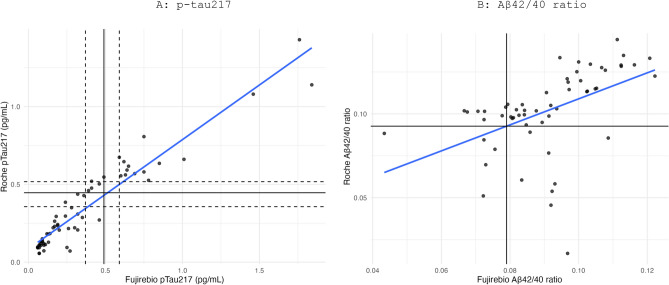
Cross-platform alignment and cutoff translation of plasma pTau217 between Roche and Fujirebio assays. (**A**) Plasma pTau217 and (**B**) plasma Aβ42/40 ratio. Scatterplots illustrate the relationship between measurements obtained using the Roche and Fujirebio assay platforms. Each point represents an individual participant with paired measurements. Solid lines indicate linear regression fits used for cross-platform bridging. Vertical lines denote published Fujirebio cut-off values, and horizontal lines represent the corresponding mapped cut-off values on the Roche scale derived from regression-based transformation. For pTau217 (**A**), the close clustering of data points around the regression line indicates strong inter-assay agreement and supports reliable translation of Fujirebio cut-offs to the Roche scale. In contrast, for the Aβ42/40 ratio (**B**), greater dispersion around the regression line reflects more limited agreement between platforms and reduced precision of cut-off translation

Receiver operating characteristic (ROC) analyses demonstrated good discrimination of biomarker-defined amyloid status for both plasma p-tau217 and the Aβ42/40 ratio (Table 4). Plasma p-tau217 measured with the Roche assay showed excellent diagnostic performance (AUC = 0.91, 95% CI 0.86–0.96), with a bootstrap-derived optimal cut-off of 0.16 pg/mL. The plasma Aβ42/40 ratio demonstrated moderate diagnostic performance (AUC = 0.79, 95% CI 0.71–0.87), with an optimal cut-off of 0.12. Cross-platform comparison between Roche and Fujirebio assays revealed strong concordance for plasma p-tau217 (ρ = 0.89; n = 84 paired samples), whereas agreement for the Aβ42/40 ratio was moderate (ρ = 0.73; n = 79). Linear regression analyses indicated a stable and approximately linear relationship between platforms for both biomarkers (Table 4). Using these regression models, published Fujirebio-based diagnostic cut-offs (summarized in Supplementary Table S2) were translated to the Roche scale. For p-tau217, the Fujirebio single cut-off of 0.49 pg/mL corresponded to a mapped Roche cut-off of 0.44 pg/mL, while the lower and upper thresholds translated to 0.35 and 0.51 pg/mL, respectively. These mapped values were closely aligned with the ROC-derived Roche cut-off, supporting good cross-platform consistency. For the Aβ42/40 ratio, the published Fujirebio cut-off of 0.08 corresponded to a mapped Roche cut-off of 0.09. Although this mapped value was directionally consistent with the ROC-derived Roche cut-off (0.12), the discrepancy between thresholds was more pronounced than for p-tau217, reflecting the lower inter-assay agreement observed for Aβ42/40. These relationships are visualized in Fig. 5A and B, which depict the cross-platform bridging between Fujirebio and Roche measurements. Overall, these findings indicate that plasma p-tau217 demonstrates robust cross-platform comparability, allowing reliable translation of established Fujirebio thresholds to the Roche assay. In contrast, the Aβ42/40 ratio showed weaker inter-assay agreement and greater dispersion, and mapped cut-offs should therefore be interpreted with greater caution.

**Table 4:** ROC and bridging results

Parameter	pTau217	Aβ42/40
*ROC analysis*		
N (ROC)	133	138
AUC (95% CI)	0.910 (0.857–0.963)	0.790 (0.712–0.869)
ROC cutoff	0.162	0.122
Cutoff 95% CI	0.137–0.196	0.119–0.127
Sensitivity	0.875	0.875
Specificity	0.885	0.656
Weighted distance	0.222	0.382
*Bridging*		
N paired	84	72
Spearman ρ	0.889	0.726
Slope	0.838	0.907
Intercept	-0.097	0.022
*Cutoff mapping*		
Single	0.490 → 0.440	0.079 → 0.094
Low	0.370 → 0.347	—
High	0.590 → 0.514	—

Receiver operating characteristic (ROC) analyses were used to derive exploratory Roche-specific thresholds for plasma biomarkers against biomarker-defined amyloid status. Published Fujirebio cutoffs were mapped onto the Roche scale using cross-platform regression models (see Supplementary Table S2). For Aβ42/40, ROC analyses included 138 participants, whereas cross-platform bridging analyses were restricted to 72 participants with paired measurements. Abbreviations: AUC, area under the curve. ROC cutoffs were derived using a sensitivity-weighted Euclidean distance approach (weight = 2) with stratified bootstrap resampling. Mapped Roche cutoffs were estimated from Fujirebio thresholds using cross-platform regression models. These mapped thresholds should be interpreted as exploratory and cohort-specific

## Discussion

This study provides an integrated analytical and clinical comparison of blood-based biomarkers across two widely used assay platforms. [17, 18] By jointly evaluating inter-assay agreement and clinical associations, we aimed to assess whether analytical concordance translates into clinically meaningful consistency.

### Principal findings

As expected, persons with Alzheimer’s disease showed a clinical and biomarker profile that included higher CDR-SB scores, lower MMSE scores, a higher prevalence of APOE ε4 and clear amyloid positivity on PET and CSF measures.

Of all biomarkers examined, plasma p-tau217 demonstrated the most consistent analytical concordance across platforms with minimal proportional bias. Consistent with this, p-tau217 showed the greatest diagnostic distinction between patients with Alzheimer’s disease and cognitively healthy controls across both assay platforms. Higher p-tau217 concentrations were also associated with greater clinical disease severity. Overall, p-tau217 demonstrated the most consistent analytical and clinical performance of all the biomarkers assessed in this study, combining high inter-assay agreement with stable diagnostic and clinical associations. However, these findings should be interpreted in the context of the present cohort and assay platforms, as assay-dependent differences in absolute values and cut-off definitions remain relevant. [10, 19, 20].

GFAP concentrations were also elevated in AD patients and showed significant associations with clinical severity. However, they exhibited lower analytical concordance and greater variability across platforms, which limited comparability and interpretability at the individual level.

In contrast, the plasma Aβ42/40 ratio showed only moderate inter-assay agreement and limited cross-platform comparability. Although the ratio differentiated AD from controls better than the individual Aβ peptides, it remained characterized by modest group separation and considerable overlap between diagnostic groups. The Aβ42/40 ratio demonstrated the strongest correspondence with CSF amyloid status across platforms, although associations remained modest overall.

From a statistical perspective, this limited separability, combined with increased dispersion across assays, renders threshold estimation inherently unstable, as small perturbations—due to measurement error or assay-dependent bias—can lead to disproportionate shifts in the estimated cutoff. ^18^ This is consistent with previous studies showing that plasma Aβ42/40 exhibits a narrow dynamic range and modest group differences, making it particularly sensitive to analytical and pre-analytical variability. [15, 16, 21–25] Importantly, high correlation between assay platforms did not imply analytical interchangeability. While p-tau217 showed close agreement between platforms, Aβ42/40 and GFAP demonstrated more pronounced assay-dependent variability and reduced interchangeability. Despite these differences, the present bridging analysis demonstrates that consistent cross-platform translation of thresholds is feasible when assay-specific calibration is applied. Age-adjusted analyses further indicated that amyloid biomarkers were less strongly influenced by age than neurodegeneration markers such as NfL. [26, 27] Taken together, these findings suggest that p-tau217 provides the most consistent analytical and clinical signal in this setting, whereas amyloid biomarkers, although biologically central to Alzheimer’s disease, are more sensitive to analytical variability and require more cautious interpretation. Importantly, analytical agreement alone does not ensure clinical interchangeability, which highlights the need to jointly evaluate both aspects.

### Comparison with previous literature

The present study extends prior single-platform investigations by providing a direct head-to-head comparison between Roche and Fujirebio assays and by linking analytical agreement to clinically relevant cut-off translation. Compared with our previous work, the ROC-derived pTau217 (0.162 pg/ml) cut-off differs modestly from the sensitivity-weighted threshold reported in a previous publication (0.182 pg/mL). [28] This difference likely reflects the incorporation of cross-platform bridging in the present analysis, which introduces regression-based calibration in addition to cohort-specific ROC optimization. The cut-off point is like that reported by Hibar et al., who proposed a two-step approach with a lower rule-out threshold of < 0.189 pg/mL, optimized for sensitivity, and a higher rule-in threshold. [29] In contrast, the present study applied a single cut-off point aimed at balanced classification, emphasizing that P-Tau217 thresholds depend on the intended clinical use. This is consistent with previous studies and consensus recommendations emphasizing that biomarker cut-offs should be interpreted in the context of the clinical setting and pre-test probability, rather than as fixed, universal values. [1, 2, 6] Similarly, the Aβ42/40 cut-off aligns with prior findings from our group based on a partially overlapping cohort, where values around 0.132 were shown to discriminate AD from controls. [18] However, it differed from the cross-platform-mapped threshold derived from published Fujirebio values (0.0807), indicating a systematic discrepancy between cohort-specific optimization and assay-harmonized cut-off translation. [2, 15, 16] From a conceptual perspective, this discrepancy may suggest that a single fixed cut-off is insufficient, and instead supports a dual-threshold approach with an intermediate “grey zone.” This is consistent with the statistical and pre-analytical uncertainties discussed above and the limited separability of the Aβ42/40 ratio. [15, 30] Amyloid biomarkers—despite their central biological role—show greater analytical variability and may require assay-specific calibration and more cautious interpretation at the individual level. Importantly, even for comparatively robust biomarkers such as p-tau217, assay-dependent differences remain, limiting the direct transferability of absolute values and cut-offs across platforms. [3–5, 10].

While the present data focus on analytical performance rather than multimarker strategies, previous work from our group demonstrated that combining p-tau and amyloid biomarkers improves diagnostic performance compared with single-marker approaches. [18] This is consistent with established biomarker frameworks such as the AT(N) classification, which emphasize the complementary roles of different biomarker domains rather than reliance on a single measure, as exemplified by CSF-based diagnostic strategies that routinely combine Aβ and p-tau biomarkers. [11, 31].

A cut-off – especially from a single marker—should not be equated with a diagnosis but rather understood as a practical threshold that depends on the population, assay, and intended clinical use (e.g. screening, rule-out, rule-in). [32, 33] These considerations also raise broader questions regarding the clinical implementation of BBBMs, including how results should be diagnostically interpreted and communicated, who should be responsible for their disclosure, and in which clinical contexts their use provides meaningful benefit for patients [6, 20].

The present findings are broadly consistent with those of recent large-scale, head-to-head comparisons of plasma biomarker assays. These comparisons include the ADNI-based study by Schindler et al., which also reported comparatively modest inter-assay correlations for plasma Aβ42/40 measures across analytical platforms. [34] One possible explanation is that ratio-based biomarkers may be particularly susceptible to analytical variability, as measurement error from both the numerator and the denominator can propagate and amplify inter-assay differences. This limits cross-platform comparability and the stability of assay-specific cut-offs. In contrast, plasma p-tau217 appears to demonstrate comparatively robust behavior across different assay technologies. Beyond the analytical comparison itself, these findings highlight the need for global harmonization and standardization strategies for blood-based biomarkers. Ongoing international efforts aim to establish reference measurement procedures, calibration standards and biologically meaningful „true values“ to improve comparability across assays and laboratories. Additionally, longitudinal assay monitoring and external quality control programmes will likely become increasingly important in order to detect potential assay drift over time, as BBBM transition into routine clinical care. Such developments may ultimately necessitate coordinated regulatory frameworks to ensure analytical reliability and clinical consistency across platforms. Importantly, the present study goes beyond a purely technical assay comparison to address the clinical implications of cross-platform variability. These include cut-off translation, the interpretation of biomarker values and assay-dependent differences in clinical applicability. These considerations are highly relevant for the future implementation of BBBM in the real-world clinical practice.

### Strengths

A key strength of this study is the direct head-to-head comparison of Roche and Fujirebio assay platforms using paired plasma samples obtained at identical time points, thereby minimizing pre-analytical variability and enabling robust assessment of inter-assay agreement. In addition, all participants underwent harmonized clinical, neuropsychological, and imaging assessments, ensuring a consistent and well-characterized reference framework across analyses. Finally, the study places particular emphasis on the stability and robustness of biomarker performance across platforms, rather than focusing solely on isolated measures of diagnostic accuracy, thereby providing a more clinically meaningful evaluation of biomarker utility.

### Limitations

This study has several limitations. First, the moderate sample size and single-center design may limit the generalizability of the findings and increase the risk of cohort-specific effects. Second, the cross-sectional nature of the analyses precludes conclusions about temporal dynamics, longitudinal trajectories, or predictive performance over time. Third, the absence of an independent external validation cohort limits the ability to confirm the robustness and transportability of the derived cut-offs across different populations and clinical settings.

Finally, the study cohort was recruited from a specialized memory clinic, which may introduce selection bias. [35, 36] In such settings, individuals typically present with more pronounced and clinically relevant cognitive impairment, whereas in the general population, memory complaints are more frequent but often nonspecific and less commonly attributable to underlying neurodegenerative disease. [37–39] Therefore, the applicability of these findings to population-based contexts may be limited. Future studies should therefore aim to validate these findings in multicenter settings with larger and more diverse populations, ideally using prospective study designs and harmonized protocols across sites.

### Implications

The present findings emphasize the importance of accounting for inter-assay variability when implementing blood-based biomarkers in clinical and research settings. Plasma p-tau217 showed comparatively stable performance across platforms, supporting its potential use in multicenter and longitudinal studies where consistent measurements are essential. However, the observed variability across biomarkers also emphasizes the need for robust harmonization strategies to enable reliable cross-platform implementation. These strategies include assay-specific calibration, normalization procedures, the use of traceable calibrators, the development of reference measurement procedures (RMPs), and candidate certified reference materials (CRMs). Without such measures, differences in measurement scales and cut-off definitions may limit comparability and hinder clinical translation. Beyond analytical considerations, these findings also emphasize the need for clear frameworks for the clinical use and interpretation of blood-based biomarkers. This includes how results are integrated into diagnostic pathways and communicated in practice.

## Conclusions

When applied in clinical practice, biomarker measurements can directly impact diagnostic classifications and treatment decisions, highlighting the importance of reliable and consistent measurements at an individual level. However, much of the current evidence is based on predicting amyloid PET positivity, i.e. a surrogate for underlying pathology rather than directly quantifiable or clinically relevant endpoints. Therefore, future studies should not only confirm analytical stability across platforms but also establish external validity across diverse populations and improve the standardization and quantification of biomarker readouts.

It is important to note that blood-based biomarkers do not measure Alzheimer’s disease as a clinical syndrome, but rather the biological features associated with its underlying pathology. Therefore, a key objective for the field is to develop an integrated framework in which markers of amyloid and tau pathology, as well as neurodegeneration, are interpreted alongside clinical context, disease stage, and longitudinal change, rather than being considered in isolation or as purely surrogate indicators.

## Supplementary Information

Below is the link to the electronic supplementary material.

Supplementary file1.

## Data Availability

The datasets generated and analyzed in the current study are not publicly available to protect participant privacy but are available from the corresponding author upon reasonable request.

## Abbreviations

AD: Alzheimer’s disease
Aβ: Amyloid beta
Aβ42: Amyloid-β42
Aβ40: Amyloid-β40
Aβ42/Aβ40: Amyloid-β42 to Amyloid-β40 ratio
AUC: Area under the curve
BMI: Body mass index
CDR-SoB: Clinical dementia rating scale sum of boxes
CL: Centiloid
CSF: Cerebrospinal fluid
FDR: False discovery rate
FU: Follow-up
GFAP: Glial fibrillary acidic protein
GDS: Geriatric depression scale
HC: Healthy controls
ICC: Intraclass correlation coefficient
INNOTEST: Immunoassay platform (Fujirebio CSF assays)
LoA: Limits of agreement
MMSE: Mini-mental state examination
MRI: Magnetic resonance imaging
NfL: Neurofilament light chain
NIA-AA: National institute on aging alzheimer’s association
PET: Positron emission tomography
p-tau: Phosphorylated tau
pTau181: Phosphorylated tau at threonine 181
pTau217: Phosphorylated tau at threonine 217
ROC: Receiver operating characteristic
SCI: Subjective cognitive impairment
SD: Standard deviation

## References

[1] Feizpour A, Doecke JD, Dore V et al (2024) Detection and staging of Alzheimer’s disease by plasma pTau217 on a high throughput immunoassay platform. EBioMedicine 109:105405. 10.1016/j.ebiom.2024.105405 39437657 10.1016/j.ebiom.2024.105405 PMC11536028

[2] Figdore DJ, Griswold M, Bornhorst JA et al (2024) Optimizing cutpoints for clinical interpretation of brain amyloid status using plasma p-tau217 immunoassays. Alzheimers Dement 20:6506–6516. 10.1002/alz.14140 39030981 10.1002/alz.14140 PMC11497693

[3] Janelidze S, Mattsson N, Palmqvist S et al (2020) Plasma P-tau181 in Alzheimer’s disease: relationship to other biomarkers, differential diagnosis, neuropathology and longitudinal progression to Alzheimer’s dementia. Nat Med 26:379–386. 10.1038/s41591-020-0755-1 32123385 10.1038/s41591-020-0755-1

[4] Kim KY, Shin KY, Chang KA (2025) Blood-based Tau as a biomarker for early detection and monitoring of Alzheimer’s disease: a systematic review and meta-analysis. Int J Mol Sci 26:20251023. 10.3390/ijms262110330 10.3390/ijms262110330 PMC12607318 41226373

[5] Pahlke S, Kahale LA, Mahinrad S et al (2025) Blood-based biomarkers for detecting Alzheimer’s disease pathology in cognitively impaired individuals within specialized care settings: a systematic review and meta-analysis. Alzheimers Dement 21:e70828. 10.1002/alz.70828 41193403 10.1002/alz.70828 PMC12590577

[6] Palmqvist S, Whitson HE, Allen LA et al (2025) Alzheimer’s Association Clinical Practice Guideline on the use of blood-based biomarkers in the diagnostic workup of suspected Alzheimer’s disease within specialized care settings. Alzheimers Dement 21:e70535. 10.1002/alz.70535 40729527 10.1002/alz.70535 PMC12306682

[7] Khalil M, Teunissen CE, Lehmann S et al (2024) Neurofilaments as biomarkers in neurological disorders - towards clinical application. Nat Rev Neurol 20:269–287. 10.1038/s41582-024-00955-x 38609644 10.1038/s41582-024-00955-x

[8] Leipp F, Vialaret J, Mohaupt P et al (2024) Glial fibrillary acidic protein in Alzheimer’s disease: a narrative review. Brain Commun 6:fcae396. 10.1093/braincomms/fcae396 39554381 10.1093/braincomms/fcae396 PMC11568389

[9] Mattsson N, Andreasson U, Zetterberg H et al (2017) Association of plasma neurofilament light with neurodegeneration in patients with Alzheimer Disease. JAMA Neurol 74:557–566. 10.1001/jamaneurol.2016.6117 28346578 10.1001/jamaneurol.2016.6117 PMC5822204

[10] Janelidze S, Bali D, Ashton NJ et al (2023) Head-to-head comparison of 10 plasma phospho-tau assays in prodromal Alzheimer’s disease. Brain 146:1592–1601. 10.1093/brain/awac333 36087307 10.1093/brain/awac333 PMC10115176

[11] Jack CR Jr., Bennett DA, Blennow K et al (2018) NIA-AA research framework: toward a biological definition of Alzheimer’s disease. Alzheimers Dement 14:535–562. 10.1016/j.jalz.2018.02.018 29653606 10.1016/j.jalz.2018.02.018 PMC5958625

[12] Klunk WE, Koeppe RA, Price JC et al (2015) The centiloid project: standardizing quantitative amyloid plaque estimation by PET. Alzheimers Dement. 10.1016/j.jalz.2014.07.003 25443857 10.1016/j.jalz.2014.07.003 PMC4300247

[13] Folstein MF, Folstein SE, McHugh PR (1975) Mini-mental state. A practical method for grading the cognitive state of patients for the clinician. J Psychiatr Res 12:189–198. 10.1016/0022-3956(75)90026-6 1202204 10.1016/0022-3956(75)90026-6

[14] Morris JC (1993) The Clinical Dementia Rating (CDR): current version and scoring rules. Neurol 43:2412–2414. 10.1212/wnl.43.11.2412-a 10.1212/wnl.43.11.2412-a 8232972

[15] Musso G, Gabelli C, Puthenparampil M et al (2025) Blood biomarkers for Alzheimer’s disease with the Lumipulse automated platform: Age-effect and clinical value interpretation. Clin Chim Acta. 10.1016/j.cca.2024.120014 39442787 10.1016/j.cca.2024.120014

[16] Bellomo G, Bayoumy S, Megaro A et al (2024) Fully automated measurement of plasma Abeta42/40 and p-tau181: analytical robustness and concordance with cerebrospinal fluid profile along the Alzheimer’s disease continuum in two independent cohorts. Alzheimers Dement. 10.1002/alz.13687 38323780 10.1002/alz.13687 PMC11032583

[17] Rauchmann BS, Brendel M, Franzmeier N et al (2022) Microglial activation and connectivity in Alzheimer disease and aging. Ann Neurol. 10.1002/ana.26465 36053756 10.1002/ana.26465

[18] Kurz C, Carli L, Gursel SU et al (2025) Plasma biomarkers of amyloid, tau & neuroinflammation in Alzheimer’s disease and corticobasal syndrome. Eur Arch Psychiatry Clin Neurosci. 10.1007/s00406-025-02013-z 40314736 10.1007/s00406-025-02013-z PMC12638345

[19] Barthelemy NR, Salvado G, Schindler SE et al (2024) Highly accurate blood test for Alzheimer’s disease is similar or superior to clinical cerebrospinal fluid tests. Nat Med. 10.1038/s41591-024-02869-z 38382645 10.1038/s41591-024-02869-z PMC11031399

[20] Karikari TK, Ashton NJ, Brinkmalm G et al (2022) Blood phospho-tau in Alzheimer disease: analysis, interpretation, and clinical utility. Nat Rev Neurol. 10.1038/s41582-022-00665-2 35585226 10.1038/s41582-022-00665-2

[21] Rabe C, Bittner T, Jethwa A et al (2023) Clinical performance and robustness evaluation of plasma amyloid-beta(42/40) prescreening. Alzheimers Dement. 10.1002/alz.12801 36150024 10.1002/alz.12801

[22] Kurz C, Stockl L, Schrurs I et al (2023) Impact of pre-analytical sample handling factors on plasma biomarkers of Alzheimer’s disease. J Neurochem. 10.1111/jnc.15757 36625424 10.1111/jnc.15757

[23] Janelidze S, Teunissen CE, Zetterberg H et al (2021) Head-to-head comparison of 8 plasma Amyloid-beta 42/40 assays in Alzheimer Disease. JAMA Neurol 78:1375–1382. 10.1001/jamaneurol.2021.3180 34542571 10.1001/jamaneurol.2021.3180 PMC8453354

[24] De Meyer S, Schaeverbeke JM, Verberk IMW et al (2020) Comparison of ELISA- and SIMOA-based quantification of plasma Abeta ratios for early detection of cerebral amyloidosis. Alzheimers Res Ther. 10.1186/s13195-020-00728-w 33278904 10.1186/s13195-020-00728-w PMC7719262

[25] Pichet Binette A, Janelidze S, Cullen N et al (2023) Confounding factors of Alzheimer’s disease plasma biomarkers and their impact on clinical performance. Alzheimers Dement 19:1403–1414. 10.1002/alz.12787 36152307 10.1002/alz.12787 PMC10499000

[26] Li W, Sun L, Yue L et al (2024) Diagnostic and predictive power of plasma proteins in Alzheimer’s disease: a cross-sectional and longitudinal study in China. Sci Rep 14:17557. 10.1038/s41598-024-66195-7 39080359 10.1038/s41598-024-66195-7 PMC11289122

[27] Smirnov DS, Ashton NJ, Blennow K et al (2022) Plasma biomarkers for Alzheimer’s disease in relation to neuropathology and cognitive change. Acta Neuropathol 143:487–503. 10.1007/s00401-022-02408-5 35195758 10.1007/s00401-022-02408-5 PMC8960664

[28] Kurz C, Tegethoff P, Hufnagel A, Perneczky, R. (2025) Validating plasma phosphorylated tau217 for clinical rule-out of cerebral amyloid pathology: evidence from a memory clinic cohort. Journal of Alzheimer´s Disease, under submission

[29] Hibar DP, Bauer A, Rabe C et al (2026) Elecsys pTau217 plasma immunoassay detection of amyloid pathology in clinical cohorts. Alzheimers Dement 22:e71009. 10.1002/alz.71009 41537338 10.1002/alz.71009 PMC12805464

[30] Ovod V, Ramsey KN, Mawuenyega KG et al (2017) Amyloid beta concentrations and stable isotope labeling kinetics of human plasma specific to central nervous system amyloidosis. Alzheimers Dement. 10.1016/j.jalz.2017.06.2266 28734653 10.1016/j.jalz.2017.06.2266 PMC5567785

[31] Hansson O, Seibyl J, Stomrud E et al (2018) CSF biomarkers of Alzheimer’s disease concord with amyloid-beta PET and predict clinical progression: A study of fully automated immunoassays in BioFINDER and ADNI cohorts. Alzheimers Dement. 10.1016/j.jalz.2018.01.010 29499171 10.1016/j.jalz.2018.01.010 PMC6119541

[32] Mielke MM, Anderson M, Ashford JW et al (2024) Recommendations for clinical implementation of blood-based biomarkers for Alzheimer’s disease. Alzheimers Dement 20:8216–8224. 10.1002/alz.14184 39351838 10.1002/alz.14184 PMC11567872

[33] Perneczky R, Quevenco FC, Hendrix J et al (2025) How can Alzheimer’s disease blood-based biomarkers reach clinical practice? Alzheimers Dement Diagn Assess Dis Monit 17:e70207. 10.1002/dad2.70207 10.1002/dad2.70207 PMC12580295 41189609

[34] Schindler SE, Petersen KK, Saef B et al (2024) Head-to-head comparison of leading blood tests for Alzheimer’s disease pathology. Alzheimers Dement 20:8074–8096. 10.1002/alz.14315 39394841 10.1002/alz.14315 PMC11567821

[35] Franzen S, Smith JE, van den Berg E et al (2022) Diversity in Alzheimer’s disease drug trials: the importance of eligibility criteria. Alzheimers Dement 18:810–823. 10.1002/alz.12433 34590409 10.1002/alz.12433 PMC8964823

[36] Leinonen A, Koponen M, Hartikainen S (2015) Systematic review: representativeness of participants in RCTs of Acetylcholinesterase Inhibitors. PLoS ONE 10:20150501. 10.1371/journal.pone.0124500 10.1371/journal.pone.0124500 PMC4416896 25933023

[37] Montejo P, Montenegro M, Fernandez MA et al (2011) Subjective memory complaints in the elderly: prevalence and influence of temporal orientation, depression and quality of life in a population-based study in the city of Madrid. Aging Ment Health 15:85–96. 10.1080/13607863.2010.501062 20924824 10.1080/13607863.2010.501062

[38] Coley N, Ousset PJ, Andrieu S et al (2008) Memory complaints to the general practitioner: data from the GuidAge study. J Nutr Health Aging 12:66S-72S. 10.1007/BF02982590 18165849 10.1007/BF02982590

[39] Yu J, Chen J, Yang L (2025) Subjective memory complaints: a conceptual analysis. J Multidiscip Healthc. 10.2147/JMDH.S518951 40666488 10.2147/JMDH.S518951 PMC12262136

