# Supplementary material for: Head-to-head comparison of neurodegeneration biomarkers across two analytical platforms in Alzheimer’s disease
Source: Aging Clin Exp Res. 2026 Jun 25;38(1):157. doi: 10.1007/s40520-026-03420-5 (PMC13314916; doi:10.1007/s40520-026-03420-5)
Supplement: Supplementary file 1 — Supplementary file1. [file 40520_2026_3420_MOESM1_ESM.docx]

# Title: Head-to-Head Comparison of Neurodegeneration Biomarkers Across Two Analytical Platforms in Alzheimer’s Disease

## SUPPLEMENT

### **Table S1:** Inclusion and Exclusion Criteria

| \| Category \| Criterion \| \| --- \| --- \| |
| --- | --- | --- |
| \| Inclusion \| Age between 55 and 85 years \| \| --- \| --- \| \| Inclusion \| Minimum of 6 years of formal education \| \| Inclusion \| Ability to provide written informed consent \| \| Inclusion \| Clinical diagnosis of Alzheimer’s disease spectrum according to NIA-AA criteria \| \| Inclusion \| Evidence of amyloid pathology in CSF (Aβ1-42/1-40 ratio < 0.055) \| \| Inclusion \| Evidence of tau pathology in CSF (pTau181 > 61 pg/mL) \| \| Inclusion \| Healthy controls: normal cognition (MMSE ≥ 28, CDR = 0) \| \| Inclusion \| Ability to complete neuropsychological testing \| \| Exclusion \| Diagnosis of non-Alzheimer neurodegenerative diseases (FTD, PSP, CBS, DLB) \| \| Exclusion \| Major psychiatric disorders (major depression, bipolar disorder, schizophrenia) \| \| Exclusion \| Significant cerebrovascular pathology on MRI \| \| Exclusion \| History of stroke or major neurological disease \| \| Exclusion \| Severe or unstable systemic illness \| \| Exclusion \| Substance abuse or alcohol dependence \| \| Exclusion \| Severe renal, hepatic, or metabolic disease affecting biomarker levels \| \| Exclusion \| Use of investigational drugs affecting amyloid or tau metabolism \| \| Exclusion \| Contraindications to MRI or lumbar puncture \| \| Exclusion \| Severe sensory impairment interfering with cognitive testing \| \| Exclusion \| Uncontrolled cardiovascular disease \| \| Exclusion \| Inability to comply with study procedures \| |

**Table S2:** Published Fujirebio-based reference thresholds for plasma biomarkers used for contextual interpretation ^1^ ^2^ ^3^ ^4^ ^5^ ^6^ ^7^ ^8^

| BBBM | FUJIREBIO CUTOFF | UNIT | COMPARISON | GROUP DEFINITION |
| --- | --- | --- | --- | --- |
| pTau217 | Low < 0.37 / High > 0.59 | pg/mL | Aβ+ vs Aβ− | Amyloid PET (Centiloid >20) |
| pTau217 | 0.49 | pg/mL | Aβ+ vs Aβ− | Amyloid PET |
| pTau181 | > 2.02 | pg/mL | AD vs HC | CSF Aβ42/40-defined amyloid status |
| Aβ42/40 ratio | < 0.0807 | ratio | AD vs HC | CSF Aβ42/40-defined amyloid status |
| NfL | Age-dependent  18–29 y: ~9.4 pg/mL  60–69 y: ~28.1 pg/mL  70–90 y: ~46.8 pg/mL | pg/mL | Reference range | Healthy individuals |
| GFAP | No established diagnostic cut-offs | pg/mL | — | — |

**Legend Table S2:** Published diagnostic cutoffs for plasma biomarkers derived from studies using Fujirebio assays. These thresholds were established based on amyloid PET imaging or clinically defined diagnostic groups and are used in the present study for cross-platform comparison and cutoff translation to the Roche assay. NfL values represent age-dependent reference ranges derived from healthy populations. GFAP currently has no established diagnostic cutoffs and was therefore treated as a continuous biomarker. For biomarkers measured on a single platform only (e.g., pTau181, NfL), published reference thresholds were not applied for classification or cross-platform analyses but are provided for contextual interpretation.

### **Table S3:** Robustness of inter-assay agreement for selected plasma biomarkers.

| Biomarker | Agreement score | Rank | n | Outliers | Spearman ρ (all/without) | PB ρ  (all/without outliers) |
| --- | --- | --- | --- | --- | --- | --- |
| Plasma p-tau217 | 1.91 | 1 | 62 | 1 | 0.914 / 0.910 | 1.121 / 1.084 |
| Plasma Aβ1-40 | 1.67 | 2 | 57 | 1 | 0.899 / 0.906 | 1.098 / 1.104 |
| Plasma Aβ1-42 | 0.31 | 3 | 59 | 1 | 0.808 / 0.845 | 0.844 / 0.857 |
| Plasma Aβ42/40 ratio | -1.60 | 4 | 57 | 1 | 0.645 / 0.675 | 0.739 / 0.779 |
| Plasma GFAP | -2.28 | 5 | 19 | 0 | 0.974 / 0.974 | 462.415 / 462.415 |

**Legend Table S3:** The table summarizes the standardized agreement ranking across plasma biomarkers together with sensitivity analyses excluding influential observations. Agreement scores were derived from standardized Spearman correlation coefficients, intraclass correlation coefficients (ICC), and Bland–Altman bias estimates. Sensitivity analyses compare Spearman correlation coefficients and Passing–Bablok slopes before and after exclusion of influential observations. Ranking was based on inter-assay agreement only. Plasma–CSF analyses are presented separately and were not included in the agreement ranking.

### **Table S4:** Effect size estimates for group differences across plasma and CSF biomarkers (AD vs. HC).

| Characteristic | Hedges g | Wilcoxon p |
| --- | --- | --- |
| N (baseline) | 86 |  |
| Roche plasma biomarkers |  |  |
| Plasma Aβ1-40 | -0.155 | 0.191 |
| Plasma Aβ1-42 | -0.782 | <0.001 |
| Plasma Aβ42/40 ratio | 0.967 | <0.001 |
| Plasma GFAP | 0.626 | <0.001 |
| Plasma NfL | 1.593 | <0.001 |
| Plasma p-tau217 | 1.453 | <0.001 |
| Plasma p-tau181 | 0.151 | 0.553 |
| Fujirebio plasma biomarkers |  |  |
| Plasma Aβ1-40 | 0.026 | 0.815 |
| Plasma Aβ1-42 | -0.473 | 0.026 |
| Plasma Aβ42/40 ratio | -1.769 | <0.001 |
| Plasma GFAP | 0.958 | <0.001 |
| Plasma p-tau217 | 2.862 | <0.001 |
| CSF Markers |  |  |
| CSF Aβ1-40 | -0.038 | 0.537 |
| CSF Aβ1-42 | -1.350 | <0.001 |
| CSF Aβ42/40 ratio | -1.099 | <0.001 |
| CSF total tau | 1.236 | <0.001 |
| CSF p-tau181 | 1.397 | <0.001 |

**Legend Table S4:** Hedges’ g effect sizes together with Wilcoxon rank-sum test p-values for the comparison between patients with Alzheimer’s disease (AD) and cognitively healthy controls (HC). Positive effect sizes indicate higher biomarker concentrations in AD compared with HC, whereas negative values indicate lower concentrations in AD. Effect sizes are reported to quantify the magnitude of group differences across biomarkers measured in plasma (Roche and Fujirebio assays) and cerebrospinal fluid (CSF). Among all biomarkers, plasma p-tau217 showed the largest effect size between AD and HC.

### **Table S5:** Cross-sectional associations of plasma biomarkers with baseline clinical severity.

| BIOMARKER | N | BETA | SE | CI LOW | CI HIGH | P | R2 |
| --- | --- | --- | --- | --- | --- | --- | --- |
| Outcome Baseline CDR-SOB | | | | | | | |
| Fujirebio plasma Aβ42/40 ratio | 46 | -48.376 | 19.355 | -87.313 | -9.440 | 0.016 | 0.284 |
| Fujirebio plasma GFAP | 46 | 0.013 | 0.004 | 0.005 | 0.021 | 0.003 | 0.329 |
| Fujirebio plasma p-tau217 | 46 | 3.425 | 0.594 | 2.230 | 4.619 | <0.001 | 0.525 |
| Roche plasma Aβ42/40 ratio | 91 | -22.543 | 10.369 | -43.143 | -1.943 | 0.032 | 0.135 |
| Roche plasma GFAP | 64 | 14.902 | 3.437 | 8.025 | 21.780 | <0.001 | 0.339 |
| Roche plasma p-tau217 | 89 | 4.281 | 0.680 | 2.929 | 5.633 | <0.001 | 0.364 |
| Outcome Baseline MMSE | | | | | | | |
| Fujirebio plasma Aβ42/40 ratio | 46 | 81.999 | 45.108 | -8.294 | 172.293 | 0.074 | 0.154 |
| Fujirebio plasma GFAP | 46 | -0.033 | 0.010 | -0.052 | -0.013 | 0.001 | 0.254 |
| Fujirebio plasma p-tau217 | 46 | -7.369 | 1.524 | -10.420 | -4.318 | <0.001 | 0.363 |
| Roche plasma Aβ42/40 ratio | 91 | 52.917 | 19.371 | 14.499 | 91.336 | 0.007 | 0.115 |
| Roche plasma GFAP | 19 | -33.999 | 6.896 | -47.788 | -20.209 | <0.001 | 0.336 |
| Roche plasma p-tau217 | 89 | -8.659 | 1.450 | -11.534 | -5.784 | <0.001 | 0.288 |

**Legend Table S5**: Linear regression models were used to assess associations between plasma biomarkers and baseline CDR-SOB and MMSE scores. Models were adjusted for age, sex, and years of education. Reported values represent regression coefficients (β) for the biomarker term, with corresponding standard errors (SE), 95% confidence intervals (CI), p-values, and model R². Higher CDR-SOB scores indicate greater clinical severity, whereas lower MMSE scores indicate worse cognitive performance.

### **Figure S1:** Adjusted associations of plasma biomarkers with neuropsychological tests**
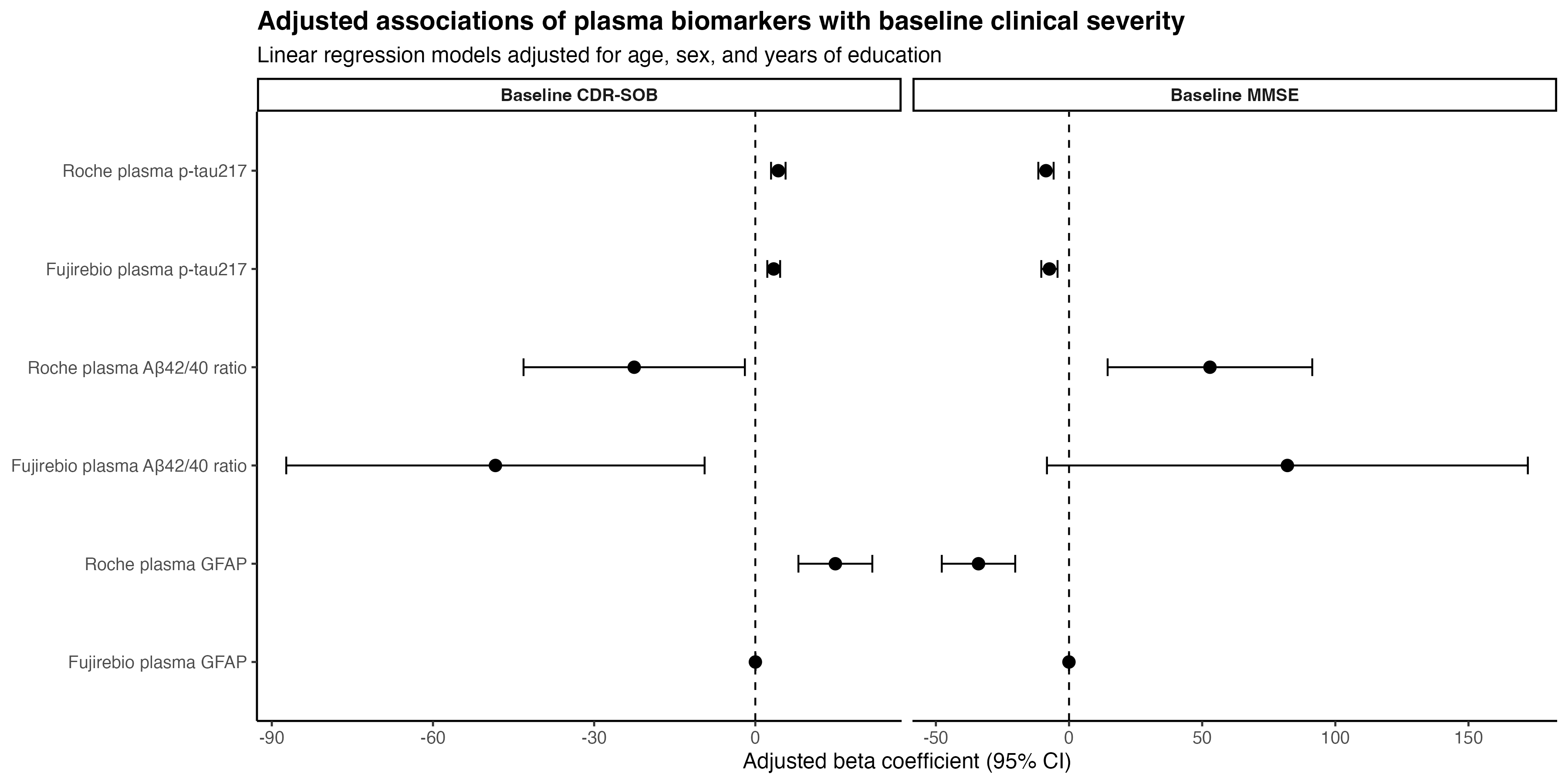
**

## LITERATURE

1. Cecchetti G, Agosta F, Rugarli G, et al. Diagnostic accuracy of automated Lumipulse plasma pTau-217 in Alzheimer's disease: a real-world study. *J Neurol* 2024; 271: 6739-6749. 20240822. DOI: 10.1007/s00415-024-12631-7.

2. Figdore DJ, Griswold M, Bornhorst JA, et al. Optimizing cutpoints for clinical interpretation of brain amyloid status using plasma p-tau217 immunoassays. *Alzheimers Dement* 2024; 20: 6506-6516. 20240719. DOI: 10.1002/alz.14140.

3. Ashton NJ, Keshavan A, Brum WS, et al. The Alzheimer's Association Global Biomarker Standardization Consortium (GBSC) plasma phospho-tau Round Robin study. *Alzheimers Dement* 2025; 21: e14508. 20250205. DOI: 10.1002/alz.14508.

4. Feizpour A, Doecke JD, Dore V, et al. Detection and staging of Alzheimer's disease by plasma pTau217 on a high throughput immunoassay platform. *EBioMedicine* 2024; 109: 105405. 20241021. DOI: 10.1016/j.ebiom.2024.105405.

5. Mondesert E, Schraen-Maschke S, Quadrio I, et al. A French multicenter analytical evaluation of the automated Lumipulse G sNfL blood assay (Fujirebio(R)) and its comparison to four other immunoassays for serum neurofilament light chain assessment in clinical settings. *Clin Chim Acta* 2025; 565: 120007. 20241023. DOI: 10.1016/j.cca.2024.120007.

6. Musso G, Gabelli C, Puthenparampil M, et al. Blood biomarkers for Alzheimer's disease with the Lumipulse automated platform: Age-effect and clinical value interpretation. *Clin Chim Acta* 2025; 565: 120014. 20241022. DOI: 10.1016/j.cca.2024.120014.

7. Simren J, Andreasson U, Gobom J, et al. Establishment of reference values for plasma neurofilament light based on healthy individuals aged 5-90 years. *Brain Commun* 2022; 4: fcac174. 20220704. DOI: 10.1093/braincomms/fcac174.

8. Bellomo G, Bayoumy S, Megaro A, et al. Fully automated measurement of plasma Abeta42/40 and p-tau181: Analytical robustness and concordance with cerebrospinal fluid profile along the Alzheimer's disease continuum in two independent cohorts. *Alzheimers Dement* 2024; 20: 2453-2468. 20240207. DOI: 10.1002/alz.13687.
